# Impact of In-Soil Ageing Effect on PLA Printed Parts Tensile Properties

**DOI:** 10.3390/polym15040862

**Published:** 2023-02-09

**Authors:** Ana P. Valerga, Severo R. Fernandez-Vidal, Franck Girot

**Affiliations:** 1 Department of Mechanical Engineering and Industrial Design, School of Engineering, University of Cadiz, Av. Universidad de Cádiz, 10, Puerto Real, 11519 Cadiz, Spain; 2 IKERBASQUE, Basque Foundation for Science, 48013 Bilbao, Spain; 3 Faculty of Engineering, University of the Basque Country, Plaza Ingeniero Torres Quevedo 1, 48013 Bilbao, Spain

**Keywords:** surface improvement, additive manufacturing, design, chemical treatment, tensile test, degradation

## Abstract

Material extrusion (MEX), more commonly known as fused deposition modelling (FDM/FFF), is one of the most widely used techniques in polymeric Additive Manufacturing (AM). This technology is increasingly present in fields such as engineering and medicine with polymeric materials, including additives of many types. Polylactic acid polymer (PLA) is one of the most widely used materials currently on the market for MEX technology. In addition to its ease of printing, it is a plastic of natural origin, biodegradable and supplants petroleum derivatives in many applications. However, the effect of ageing on the mechanical properties of PLA are still to be evaluated and understood. The main objective of this work is to investigate the effects of ageing of PLA samples on the tensile properties. To investigate the effect of ageing, the samples were tested periodically after exposure to fertilized soil for a period up to 6 months. In addition, some of the samples were chemically pre-treated to improve the surface quality, and the effect of ageing on the treated and untreated samples was also evaluated. This study showed that ultimate strength decreased with ageing from 46 to 36 MPa (22%), and it increased with treatment time in high percentages (even 40%) depending on the time of immersion in the solvent. However, this effect of the chemical treatment gradually disappeared, with the exception of the surface improvement obtained.

## 1. Introduction

Industry 4.0 is a global set of emerging technologies that establish a new industrial perspective. It has radically transformed production processes and systems with the adoption of enabling technologies such as additive manufacturing (AM). In this context, additive manufacturing offers agility and customer focus in any industry, enabling multi-material, free manufacturing and flexibility [1,2].

In the early 1990s, the inventor Stratasys developed a new method for additive manufacturing or 3D printing: Fused Filament Fabrication (FFF) or Fused Deposition Modelling (FDM). FDM technology is based on the creation of parts from the deposition of layers of extruded thermoplastic material on a heated platform [3,4]. It is currently the most widely used additive manufacturing process and is present in a wide range of sectors. Furthermore, it has been demonstrated that 3D printing technology could reduce labor by 80%, construction materials by 60% and as a result achieve an overall cost reduction of up to 30%. These benefits are thanks to the integration of Industry 4.0 and new emerging technologies [5].

However, it has one major disadvantage: the use of polymers, with a total annual production of 400 million tons of polymers in 2021 [6,7]. This would not be so worrying if these materials did not take hundreds of years to decompose. During this long degradation process, they coexist alongside other species in oceans and forests and greatly hinder their life cycle. In addition, this waste, exposed over a long period of time to ultraviolet radiation, breaks down into small fragments of less than 5 mm, which are called microplastics. More than 51 trillion of these particles are currently floating in the seas and oceans, making it easier for marine animals to consume, and thus, they reach food and products consumed by humans. The vast majority of plastics are obtained from fossil hydrocarbons and petroleum derivatives and are not recyclable materials, inducing dramatic ecological consequence from their massive use. One of the proposed solutions is to drastically reduce the consumption of plastics or to improve recycling systems, since of the 6 billion tons of plastics converted into waste, only 9% has been recycled [8].

For this purpose, the study of biodegradable polymers has shown a growing interest in this technology. Polylactic acid (PLA) is one of the most widely used materials that continues to be studied today. PLA is a natural polymer derived from renewable sources such as starch, a large carbohydrate that plants synthesize during photosynthesis. Bioplastics produced from this polymer have the characteristic of being a resin that can be injected, extruded and thermoformed [9]. Thus, PLA has played a central role in replacing fossil-based polymers for certain applications as a fully aliphatic polymer [10,11]. As a polymer, PLA is considered a promising alternative to reduce the municipal solid waste (MSW) disposal problem.

Due to its biodegradability, barrier properties and biocompatibility, numerous applications have been found with this biopolymer, as it exhibits an unusually wide range of properties (mechanical properties in the same range as petrochemical polymers, except for low elongation [12]), from the amorphous to the crystalline state—properties that can be achieved by manipulating the mixtures between D- and L-isomers, molecular weights, copolymerization and material treatments [13].

Furthermore, the changes in properties during PLA biodegradation are very specific [14]. Indeed, the biodegradation of PLA proceeds more slowly compared to other similar plastics, probably due to the higher stability of PLA towards hydrolysis [15]. Due to degradation, the elongation at break decreases drastically, while the tensile strength, Young’s modulus and impact strength do not change substantially [16]. These aspects, however, have been vaguely studied in parts manufactured with FDM technology.

The failure processes of FDM-manufactured polymers are defined by the structural irregularity of the system, which is characterized by the presence of interfacial and interlayer bonds. The individual events involved in the development of failure and fracture can be too complicated to describe, especially if they have a complex microstructure such as PLA. Therefore, the study of the monolayers is proposed in this work.

Consequently, this work aims to analyze the degradation of PLA parts manufactured by FDM exposed to fertilized soil over a period of 6 months. For this purpose, monolayers were used. These were manufactured, and some of them were treated with a chemical agent to improve their surface quality. Subsequently, tensile tests were carried out periodically, and the effect of the ageing of the material on the mechanical properties was evaluated in treated and untreated parts.

## 2. Materials and Methods

**Figure 1:**
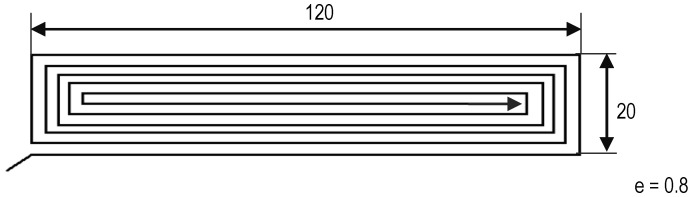
Dimensions and trajectories used for monolayer samples.

An open-source test bench with a 0.4 mm diameter nozzle was used for processing specimens. Monolayers of FFF World’s Polylactic Acid (PLA) were fabricated, whose geometry and trajectories are shown in Figure 1 [17]. The monolayer thickness was 0.8 mm. The material specifications given by the manufacturer are listed in Table 1. This FFF World’s (Alicante, Spain) PLA was obtained from Natureworks. It is a mixture of both PLLA and PDLA (meso-lactide, PDLLA). Commercial PLA filament for FDM is usually an L-rich mixture of these isomers. 

The manufacturing parameters used are given in Table 2.

Once the parts were manufactured, they were weighed and immersed according to the variable immersion time [Ti] for 1 to 30 s in an organic solvent, chloroform (CHCl_3_, Laboquimia, Logroño, Spain), as shown in Table 3. This solvent has been used in other studies, showing a favorable behavior for the improvement of the surface quality of the parts manufactured with FDM [18,19]. After the treatment, specimens were weighed again. The parts were dried in ARL-0680 ESPEC climatic chamber (ESPEC, Osaka, Japan) before ageing.

Five samples of each type were tested the day after the treatments and considered as reference (ageing time = 0). Subsequently, the remaining samples were deposited in fertile soil with the characteristics shown in Table 4 for a period of 6 months. The samples were tested periodically according to the variable ageing time [At] to estimate the evolution of the degradation on the material. A factorial study of Ti and At with values included in Table 3 was carried out.

A contact profilometer, Mahr Perthometer PGK 120 (Mahr GmbH, Göttingen, Germany), was used for microgeometric characterization. Also, image analysis was performed by a Hitachi SU1510 Scanning Electron Microscope (SEM, Hitachi, Tokyo, Japan) at low vacuum mode at 10 kV. A backscattered-electron (BSE) detector was used for imaging the surface of the samples.

Mechanical tests were performed with the universal testing machine Shimadzu^®^ AG-X (Shimadzu, Kyoto, Japan). These tensile tests were achieved with a constant testing speed of 2 mm/s, as suggested in ISO 527 for standardization for molding and extrusion polymers [20]. These tests were carried out under conditions of temperature and humidity similar to those of service parts (25 °C and 50%). The aim of this experiment was to evaluate the efficiency of the surface quality improvement method used (solvent immersion) and how it affects the mechanical properties of the treated parts after a period of time.

## 3. Results

**Figure 2:**
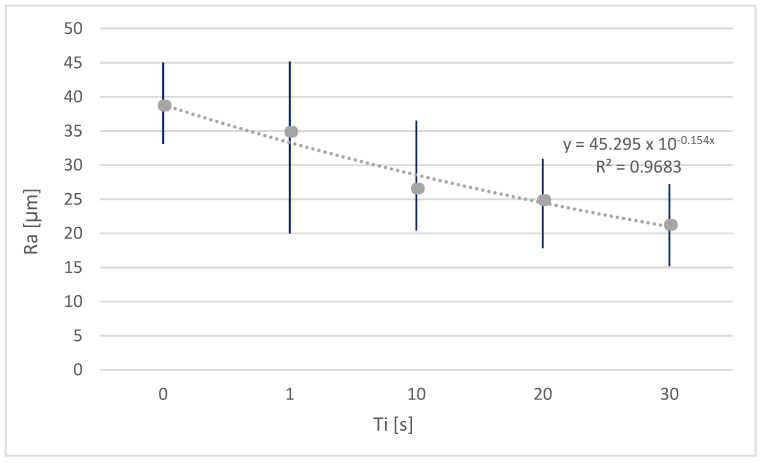
Arithmetic mean roughness (Ra) as a function of immersion time in chloroform.

Chemical treatments have been optimized in previous works for the improvement of surface quality [13]. In this work, different immersion times [Ti] in a single solvent (chloroform) have been evaluated. Figure 2 illustrates that the longer the immersion time, the more marked the improvement—the trend of which follows an exponential function. This improvement may be due to the modification of the material by the treatments. Thus, the material becomes viscous or semifluid as it absorbs the volatile solvent. This affects the peaks and dips, and under the effects of fluid surface tension and gravity, these become largely homogenized, resulting in lower peaks and fuller dips. As time passes, a dynamic equilibrium is achieved, resulting in a smoother surface, which should lead to an improvement in the surface quality of the parts obtained. However, it has been reported in the literature that with very long immersion times, the parts begin to show significant deviations from their original geometry. For this reason, the limit was set at 30 s [21]. 

If the surface improvement is compared with other works in which the same procedure was applied, a much lower improvement is observed for the same period of time. This is probably due to the quantity and distribution of the isomers that form the PLA in each case, which cause a greater or lesser probability of crystallite formation. However, this improvement in surface roughness for such a short and simple treatment is very satisfactory.

After 180 days, the surface quality was intact. However, chemically treated samples shrunk significantly. This effect is associated with the fact that the initially treated samples expanded due to the absorption of the chlorine present in the solvent used. 

There are several modes of fracture in polymeric materials, namely interlaminar delamination or interlayer fracture, intralaminar or interfacial cracking or fracture, and crack propagation through the pores generated by the process. Typically, the failure processes in all are time dependent, reflecting, at least in part, the viscoelastic nature of the mechanical response of the polymer.

**Figure 3:**
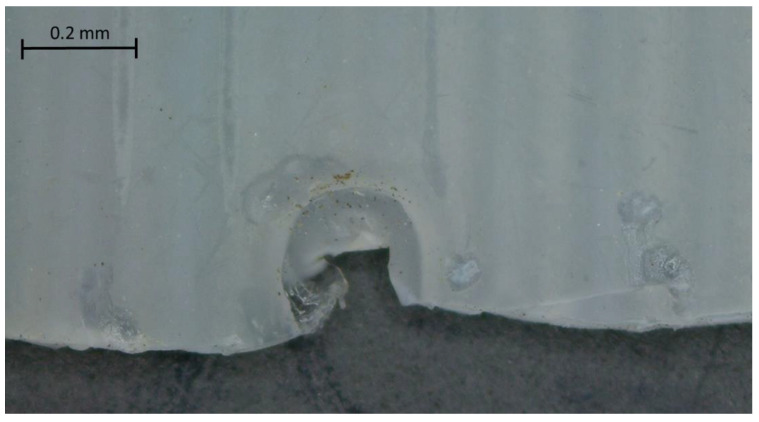
Chlorine-swollen pore causing premature failure.

The main cause of failure in polymers is structural damage on a microscopic scale, such as microcracks. These are the result of impacts and internal stresses and are dangerous due to their undetectable nature and also due to the induced fragmentation of the structure, leading to reduced mechanical properties such as strength, stiffness and corrosion. Adding the layered manufacturing process increases these types of defects from the manufacturing process itself. These internal defects not only decrease the performance of the material, but also serve as catalysts for other damage, such as macrocracks, moisture swelling and corrosion. This microcrack, swollen by the action of chlorine, can be seen in Figure 3. The halogenated component of this solvent, chlorine, is by nature a non-metal located in group VII of the periodic table. These substances have a defined partial pressure that increases with the amount of volatilized chlorine gas, which, due to thermodynamic effects (chemical potential), tries to escape to the outside and meet the pores of the polymer, which expands due to the amount of chlorine atoms that try to escape from the surface (Figure 3). In other words, the chlorine tries to dissolve the polymer material by invading its structure and swelling it. This causes a volume increase in the samples. The gas tries to escape through the largest area of the sample, which was also placed horizontally for drying over a long period of time, which is why the thickness was first increased. However, this softening of the material causes the large voids (interfacial and interlayer cavities) to stick together. 

**Figure 4:**
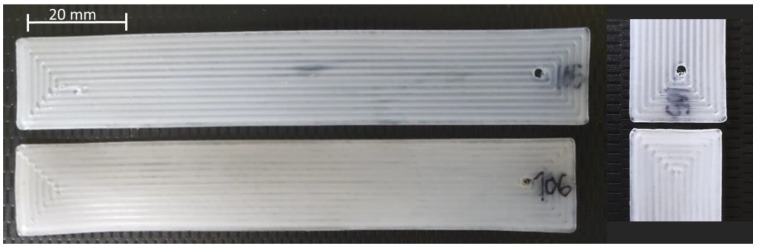
Dimensional compaction of a sample treated in chloroform for 30 s before and after ageing time.

When parts are subjected to mechanical testing, cracks initiate mainly on the surfaces of the parts and propagate through those microcracks or through bad interlayer and inter-face joints (between deposited filaments) that originate small pores in the interior. These pores are the ones that appear in greater proportion in solvent-treated samples, which causes greater crack propagation. Over time, this volatile solvent evaporates, causing the compaction of the samples to increase. This leads to a decrease in their volume, as can be seen in some of their dimensions in Figure 4.

For all polymeric materials, the theoretical strength of the interfacial bonds is several orders of magnitude greater than the measured fracture stresses. This is accentuated in Additive Manufacturing processes, where in addition to crack defects that can produce significant local stress concentrations, there is a very strong influence from the interface paths and bonds that cause further concentrations. Therefore, by the nature of the manufacturing process, the material is not subjected to uniform stresses. A disproportionate amount of load is carried when stress is applied to a polymer sample, which can be sufficient to overcome the bond strength. This non-uniformity in stress is much more pronounced in amorphous polymers such as PLA, whereas treated parts exhibiting higher crystallinity should distribute stress more evenly. However, this does not occur as expected. Chain scission can occur at relatively low stresses in cross-linked or highly aligned polymers, but the mechanical response of isotropic polymers with a low cross-link density is governed by secondary bonds at low stresses. This means that many polymers yield before fracture at high stresses.

Highly cross-linked polymers are incapable of large-scale viscoelastic deformation. Molecular chains overcome van der Waals forces, but it does not apply to cross-linked polymers, where the primary bonds between chain segments must be broken for these materials to deform [22]. For this reason, the deformation measured in these pieces presents a large dispersion and is not analyzed in this work.

**Figure 5:**
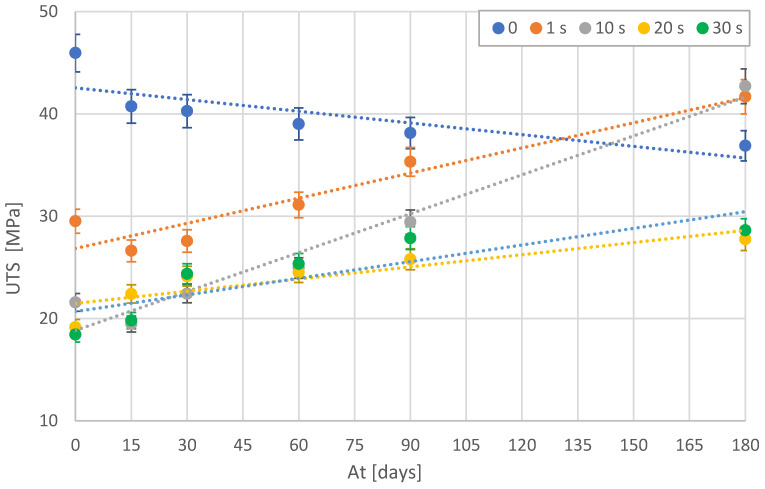
Average ultimate tensile strength (UTS) of treated and untreated samples as a function of ageing time [At].

Ultimate fracture occurs in a zone where individual fibrils break and generate a microcrack, which is unstable if the redistributed stress is sufficient to break one or more neighboring fibrils. Fracture in a crack zone is usually initiated by particles from areas of missing material or impurities (inorganic dust trapped in the polymer) [23]. The treated material, due to the volatilization of chlorine, has more impurities that cause a premature failure of the material. Hence, the more the samples are treated, the lower the tensile strength [T] is achieved (Figure 5).

After the linear fitting of the data in Figure 5, a minimum correlation of 0.66 and a maximum of 0.96 was obtained, suggesting that there is a correlation between the tensile strength and the degradation of both a treated and untreated PLA part. The regression lines slope quite steeply, changing the final value by as much as 50% from the initial value, suggesting quite a significant change in the final tensile strength. The standard deviations are around 4% of the values.

**Figure 6:**
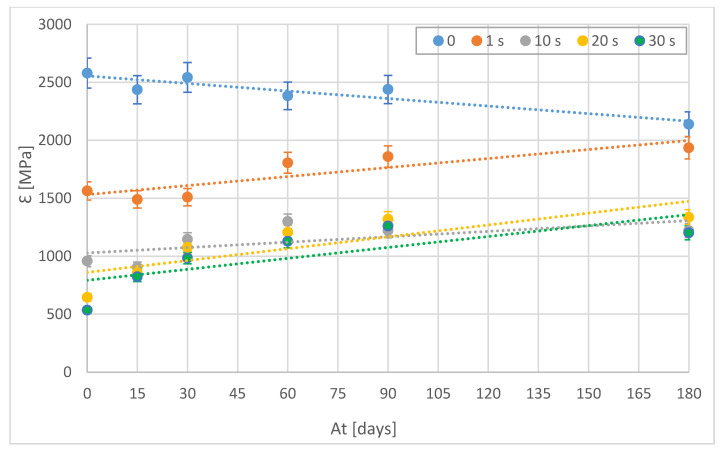
Yield strength (ε) of treated and untreated samples as a function of ageing time [At].

On the other hand, the elastic modulus (ε) behaves similarly to the maximum stress with similar settings (Figure 6). Thus, untreated samples lose stiffness over time. In contrast, the treated samples, which initially become more elastic, approach the values of the untreated samples over time. This indicates a loss of the impact of the treatment on the material over time.

**Figure 7:**
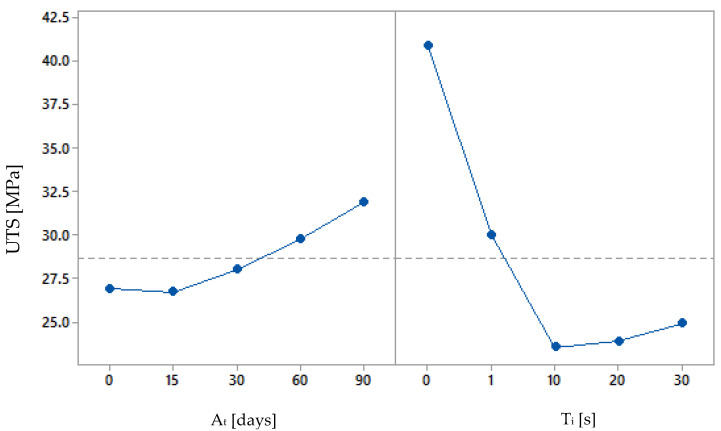
Main effects of ageing effect [At] and immersion time [Ti] on the average ultimate tensile stress [UTS] of the samples.

**Figure 8:**
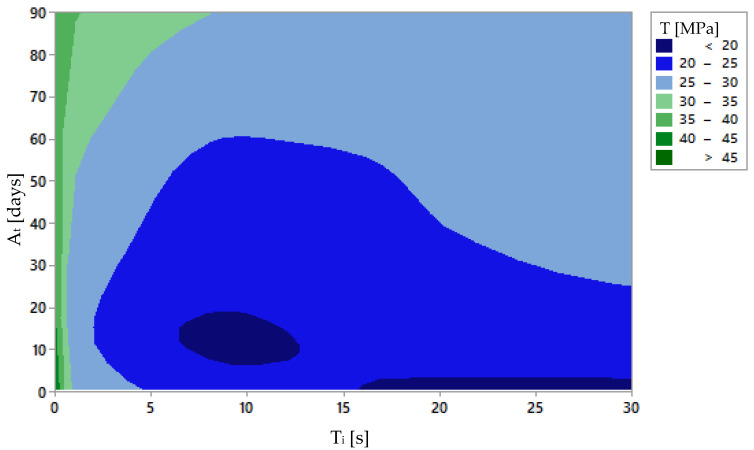
Contour graphic of immersion time [Ti] and ageing effect [At] on the average ultimate tensile stress of the samples.

As can be seen in Figure 5, Figure 6, Figure 7 and Figure 8, in general, the mechanical properties of the PLA material degrade with ageing. It can be observed that the degradation process is systematic and practically linear. However, it is interesting to observe how the solvent-treated samples behave in a completely opposite way. These samples were structurally modified by the action of chlorine [23]. Everything seems to indicate that this modification is disappearing with the passage of time, returning to its initial state. Interestingly, the surface texture increased considerably and remains in perfect condition due to the thin layer formed by the action of the solvent. Therefore, over time, these treated samples tend to have the same mechanical strength as the original parts, but with a considerable surface improvement.

Other authors have studied the degradation process, taking into account different layer thicknesses, and came to the conclusion that the higher the layer height, the greater the mechanical degradation due to the higher proportion of air voids or porosities [24]. These voids, in the case of treatments, favor the expansion along the whole sample and the homogeneous application of the treatment. In turn, they disappear over time, and the sample is compressed. This is the reason for the volume reduction mentioned above. It is estimated that the thicker the layer, the more this will occur. However, it would be necessary to analyze in more detail how it affects multilayer parts if the orientations or path distributions are modified.

The average decrease in strength in all the untreated samples was around 25%, while in the chemically treated samples, an increase in strength of more than 50% was obtained in all cases, reaching 100% in some of them. This phenomenon should be examined in other environments and additionally with PLA filaments from different manufacturers. 

Figure 7 and Figure 8 show the main effect plot and the contour plot of the tensile strength results. They are interesting as they show that the solvent worsens the tensile strength, but the tensile strength improves faster, proportionally, over time. It is concluded that after a longer period of time, samples treated for a longer immersion period will also reach the mechanical capacity of the others. However, the best ratio of surface quality to ageing time has to be found.

**Figure 9:**
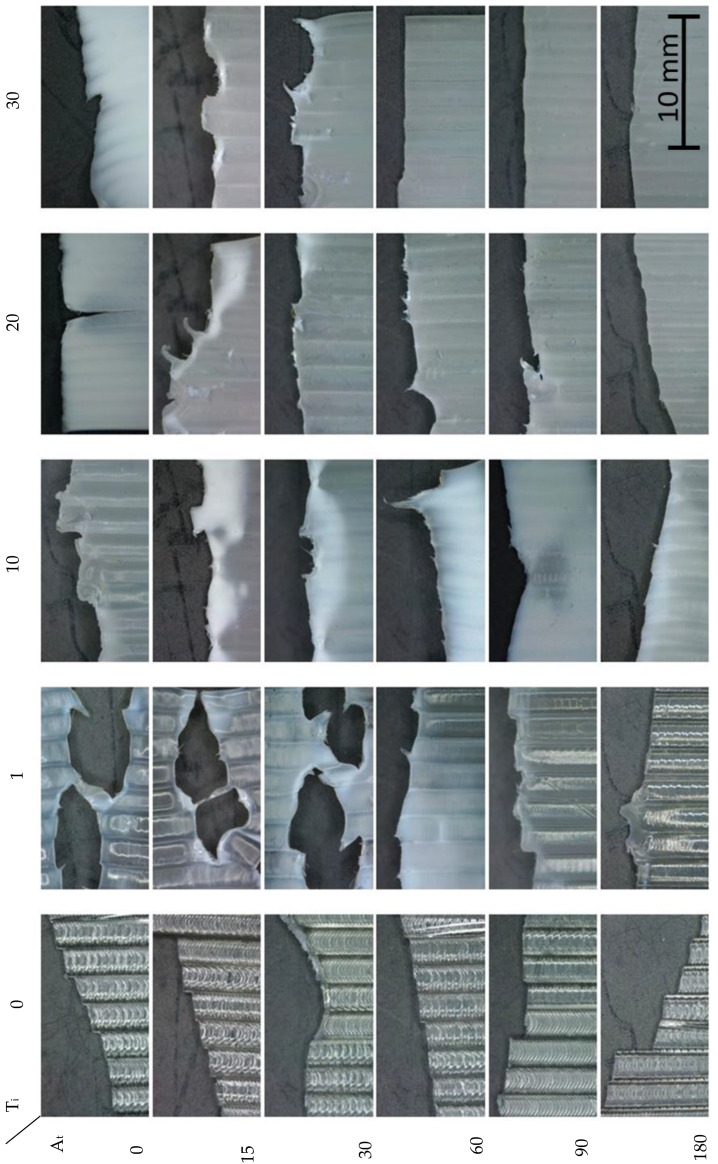
Example of fracture in specimens for each test type.

No plastic deformation was observed in the chemically untreated specimens [25]. If we analyze any of the untreated specimens, the displacement-controlled loading leads to an almost linear increase until the maximum load is reached, regardless of whether it has been aged; this can be seen in the images in Figure 9. 

In PLA, a creep zone usually forms at the crack tip. However, shear creep produces a Dugdale-type band creep zone ahead of the crack tip. Of the two creep mechanisms in the untreated PLA, crazing is rather more likely ahead of the crack tip, due to the triaxle stress state. For this reason, these samples show a more brittle and continuous fracture (Figure 9). However, in some cases, each strand broke individually, resulting in a crack discontinuity. The treated samples appear to show the fibril breakage mechanism. For this reason, much more pronounced stress concentrations vaguely appear in different areas, breaking the sample into several zones. This is also one of the main reasons for the much lower stresses supported [26].

A common problem in polymers under stress in a chemical environment is stress corrosion cracking (SCC), for example in polyolefin pipes, where it is often observed as a colony of microcracks within a degraded polymer surface layer. This can resemble what occurs in parts that have been chemically treated for surface improvement and often results in multiple crack initiations due to individual pore growth.

The ageing effect is also observed in the type of fracture. The high elasticity obtained in solvent-treated samples is gradually lost over time. Thus, the continuous and brittle fracture of the untreated samples is obtained again, although it is continuous. The continuity of the fracture is due to the inter-face bond that is built up after the application of solvents.

## 4. Conclusions

The surface quality of parts manufactured by fused deposition modelling using chemical baths has been improved. Specifically, chloroform has been used as a solvent to improve the roughness (Ra) by almost 50% in only 30 s of treatment.

The impact of ageing on the mechanical properties of FDM printed parts with PLA was analyzed by experimental tests. It was observed that PLA parts degrade with ageing on the ground, and this was manifested in a decrease in tensile mechanical properties. The trend continued to decrease during the whole period of the experiment (6 months).

It was also interesting to see the worsening of the mechanical strength when the parts were subjected to chemical baths. However, these parts increased their breaking strength value as time went by when they were exposed to a supposed degradation. This seems to indicate that PLA, whose structure was affected by the baths, recovers its properties. Although the treatment of aged parts improved the strength of the specimens, it is interesting to note that the tendency was to obtain a value similar to that of untreated and unaged specimens. This indicates that the treatment was clearly lost. Thus, less rough parts can be obtained, and after a few months they will have recovered the mechanical properties lost during the solvent bath. 

The influence of immersion time on the surface improvement as well as on the tensile strength, and the influence of ageing time on the mechanical properties have been demonstrated.

The experiments were conducted on standardized parts for tensile tests to give an idea of the part’s behavior evolution with this type of process and to be able to compare it with other studies. Furthermore, this study leads to the consideration that the moment of testing throughout the life cycle of a part is decisive; thus, the authors consider this to be one more variable to be considered for all studies, as the material or layer height is indicated.

## Figures and Tables

**Table 1:** Material specifications.

	Tensile Strength at Yield	Strain at Yield	Tensile Modulus	Melting Temperature
Value	60 Mpa	4%	3120 Mpa	115 ± 35 °C

**Table 2:** Values of manufacturing parameters.

	Speed [mm/s]	Overlap [%]	Nozzle Temperature [°C]	Bed Temperature [°C]	Retraction [mm and mm/s]
Value	20	55	220	65	1.7 and 15

**Table 3:** Factors for trials.

Ti [s]	0	1	10	20	30
At [Days]					
0	5	5	5	5	5
15	5	5	5	5	5
30	5	5	5	5	5
45	5	5	5	5	5
60	5	5	5	5	5
90	5	5	5	5	5
180	5	5	5	5	5

**Table 4:** Soil characteristics.

	Organic Material [%sms]	PH	Humidity	C.E. [mS/cm]	Relation C/N	Nutrient Content [%]		
						N	P	K
Value	30.2	7.5	28.9	1.5–2	15–25	1.11	0.54	1.43

## Data Availability

The data that support the findings of this study are available from the corresponding author upon reasonable request. Also, there are some images uploaded on Open Science Framework.
